# ^18^F-Fluorodeoxyglucose Positron Emission Tomography/Computed Tomography for Assessing Treatment Response of Pulmonary Multidrug-Resistant Tuberculosis

**DOI:** 10.3390/jcm7120559

**Published:** 2018-12-17

**Authors:** Joon Young Choi, Byung Woo Jhun, Seung Hyup Hyun, Myung Jin Chung, Won-Jung Koh

**Affiliations:** 1 Department of Nuclear Medicine, Samsung Medical Center, Sungkyunkwan University School of Medicine, 81 Irwon-ro, Gangnam-gu, Seoul 06351, Korea; jynm.choi@samsung.com (J.Y.C.); shnm.hyun@samsung.com (S.H.H.); 2 Division of Pulmonary and Critical Care Medicine, Department of Medicine, Samsung Medical Center, Sungkyunkwan University School of Medicine, 81 Irwon-ro, Gangnamgu, Seoul 06351, Korea; byungwoo.jhun@gmail.com; 3 Department of Radiology, Samsung Medical Center, Sungkyunkwan University School of Medicine, 81 Irwon-ro, Gangnam-gu, Seoul 06351, Korea; mjchung@skku.edu

**Keywords:** Pulmonary tuberculosis, Multidrug-resistant tuberculosis, ^18^F-FDG, PET/CT, Therapeutic response

## Abstract

Background: The purpose of this prospective study was to evaluate the role of ^18^F-Fluorodeoxyglucose (^18^F-FDG) positron emission tomography/computed tomography (PET/CT) for assessing treatment response in patients with pulmonary multidrug-resistant tuberculosis (MDR-TB). Methods: The study subjects were four patients diagnosed with pulmonary MDR-TB who underwent MDR-TB treatment and serial ^18^F-FDG PET/CT at baseline and 6 and 12 months after treatment. The highest lung maximum standardized uptake value (SUV_max_), average SUV_mean_ (average of all hypermetabolic parenchymal lesions), total metabolic lung volume (TMLV, sum of metabolic volumes from the hypermetabolic parenchymal lesions), and total lung glycolysis (TLG, sum of lesion glycolysis from the hypermetabolic parenchymal lesions) were determined as representative quantitative PET parameters for each patient. Results: All patients except one had negative sputum culture conversion after one month of treatment and achieved successful treatment outcomes. Baseline TMLV and TLG PET parameters were much higher in the single patient with treatment failure than in the remaining three patients with treatment success. No other PET parameters at baseline or follow-up were associated with the treatment results. Conclusions: Pretreatment volume-based ^18^F-FDG PET/CT lung parameters were associated with the final therapeutic response in patients with pulmonary MDR-TB. Our preliminary results warrant a larger study.

## 1. Introduction

Multidrug-resistant tuberculosis (MDR-TB), which is caused by *Mycobacterium tuberculosis* strains that are resistant to both isoniazid and rifampin, is a large public health threat. The majority of MDR-TB cases have unsatisfactory outcomes; there is a treatment success rate of approximately 60% despite the use of second-line anti-TB drugs for 18–24 months [1,2]. Historically, MDR-TB treatment response is monitored by microbiological examination of sputum-negative culture conversion [3]. However, sputum examinations can be affected by subjective symptoms, difficulty in patient sputum expectoration, monitoring intervals, and microbiology methods [4]. Additionally, the value of sputum examinations for predicting TB relapse is low [5]. Because most MDR-TB patients with therapeutic responses achieve culture conversion within three months after starting treatment, monitoring during the remaining scheduled treatment period using standard clinical or radiological tools is relatively limited [6].

^18^F-fluorodeoxyglucose (^18^F-FDG) positron emission tomography/computed tomography (PET/CT) was originally used to evaluate malignancies. However, this method has been proposed for the diagnosis and monitoring of treatment response of infectious diseases, including TB [7,8]. Sites of active inflammation show intense glucose hypermetabolism, and neutrophils and activated macrophages at infection sites may be responsible for the accumulation of ^18^F-FDG [9]. For assessment of ^18^F-FDG uptake, qualitative interpretation by visual inspection and quantitative analysis are used [7]. Standardized uptake value (SUV) is the most commonly used tool for assessing ^18^F-FDG uptake [7]. Volumetric ^18^F-FDG uptake measurements such as metabolic volume and lesion glycolysis have been introduced for accurate assessment of ^18^F-FDG uptake [10]. Metabolic volume is the volume of the lesion with active ^18^F-FDG uptake, and lesion glycolysis is calculated by multiplying the lesion SUV_mean_ by the metabolic volume, which represents both lesion size and extent of ^18^F-FDG uptake [10]. 

Intense glucose hypermetabolism and high ^18^F-FDG uptake have been observed in patients with TB, and a few previous studies have shown the utility of ^18^F-FDG PET/CT for determining TB disease activity or evaluating therapeutic response [11,12,13,14]. To date, data are limited on the utility of ^18^F-FDG PET/CT for assessing treatment response in patients with MDR-TB. All previous studies used qualitative interpretation and SUV measurements, but did not include volumetric ^18^F-FDG uptake measurements such as metabolic volume and lesion glycolysis. Therefore, the purpose of this prospective preliminary study is to evaluate the role of ^18^F-FDG PET/CT for assessing treatment response in patients with MDR-TB in the lung.

## 2. Experimental Section

### 2.1. Subjects

The subjects were four patients diagnosed with pulmonary MDR-TB from March 2015 to October 2015 at the Samsung Medical Center. Pulmonary MDR-TB was confirmed by sputum culture and drug susceptibility tests in all patients. This prospective study was approved by the Institutional Review Board of our institute (IRB No: 2014-08-113-012), and written informed consent was obtained from all participants. 

### 2.2. ^18^F-FDG PET/CT

^18^F-FDG PET/CT images were obtained at the start of the MDR-TB treatment and at 6 and 12 months after second-line MDR-TB treatment. All patients fasted for at least 6 h before PET/CT studies. Their blood glucose level was required to be less than 200 mg/dL. Chest PET and unenhanced CT images were acquired using an integrated PET/CT scanner (Discovery STE, GE Healthcare, Milwaukee, WI, USA). Chest CT images were obtained with shallow breathing using a 16-slice helical CT with 60 to 200 mA adjusted to patient body weight at 120 kVp and a section thickness of 3.3 mm. After CT and 60 min after intravenous injection of ^18^F-FDG (5.0 MBq/kg), an emission scan was performed from the lower neck to the upper abdomen at 2.5 min per frame intervals in three-dimensional mode. PET images were reconstructed using CT for attenuation correction with an ordered-subsets expectation maximization algorithm (20 subsets, two iterations) and a voxel size of 3.9 × 3.9 × 3.3 mm. SUV was normalized to patient body weight.

Two nuclear physicians unaware of clinical data interpreted all PET/CT findings by consensus. Volume-based assessment by ^18^F-FDG PET/CT was performed using commercially available medical imaging software (MIM version 6.4, MIM Software Inc., Cleveland, OH, USA). We set the volume of interest over the entire lung to encompass disease involving the whole lung parenchyma, and the software segmented the hypermetabolic parenchymal lesions above the SUV threshold of 2.5 in both lungs. Maximum SUV (SUV_max_), average SUV (SUV_avg_), and metabolic volume of the segmented hypermetabolic parenchymal lesions were measured. Lesion glycolysis of each hypermetabolic parenchymal lesion was acquired as the product of SUV_avg_ and metabolic volume. The highest SUV_max_, mean SUV_avg_ (mean of SUV_avg_ from all hypermetabolic parenchymal lesions), total metabolic lung volume (TMLV, sum of metabolic volumes from the hypermetabolic parenchymal lesions), and total lung glycolysis (TLG, sum of lesion glycolysis from the hypermetabolic parenchymal lesions) were determined as representative metabolic PET parameters for each patient.

### 2.3. MDR-TB Treatment and Clinical Follow-Up

All patients diagnosed with MDR-TB were treated with individualized second-line regimens for 18–24 months, with at least four effective drugs during the 6–8 months of the intensive phase, as per World Health Organization guidelines [15]. If the isolates had additional resistance to fluoroquinolone, linezolid and bedaquiline were added to the treatment regimens. Sputum smear examinations and cultures were performed monthly for the first 6 months and then at 2- to 3-month intervals until the end of treatment. Treatment success was defined as the completion of treatment without evidence of treatment failure and three or more consecutive negative cultures performed at least 30 days apart after the intensive phase [15]. Treatment failure was defined as a lack of conversion by the end of the intensive phase or bacteriological reversion in the continuation phase after the conversion to negative [15].

## 3. Results

Four patients were included in the study, and three of them were female (Table 1). The age of the study patients ranged from 35 (patient 1) to 61 (patient 4) years. All patients met the definition of MDR-TB, and patients 2 and 3 had MDR-TB with additional resistance to fluoroquinolone. Patients 1 and 2 had positive acid-fast bacilli smear results at diagnosis. Body mass index was highest in patient 4 (25.0 kg/m^2^) and lowest in patient 2 (15.1 kg/m^2^). Patient 2 was previously treated for MDR-TB using second line anti-TB drugs for 23 months and underwent pneumonectomy, approximately eight years before study enrollment. Patients 1 and 4 were previously treated for TB lymphadenitis and drug-susceptible pulmonary TB, respectively. Cavitary lung lesions were observed in patients 1–3. Infiltrations suggestive of active pulmonary TB were mainly observed in the upper lobes of patients 2–4, with the involvement of the entire right lung in patient 2. Patient 1 had right lower lobe infiltration. Serum erythrocyte sedimentation rate (ESR) was lowest in patient 4, but there was no notable difference in the baseline characteristics of study patients.

Treatment outcomes for MDR-TB and serial changes of PET/CT parameters are shown in Table 2. All patients were treated with a combination of second-line MDR-TB regimens, such as pyrazinamide, injectable aminoglycosides, fluoroquinolone, prothionamide, cycloserine, para-aminosalicylic acid, or clofazimine. Linezolid and bedaquiline were included in the treatment regimens in patients 2 and 3. Three of the four patients had negative sputum culture conversion after one month of treatment and achieved final treatment success (patients 1, 3, and 4). These three patients showed clinico-radiological improvement, and cavitary lesions in patients 1 and 3 were resolved. The treatment durations of the three patients were 20.1, 21.8, and 19.8 months, respectively. The three successfully treated patients were followed for 17.0, 20.8, and 6.7 months, respectively, after treatment without recurrence. However, patient 2 did not achieve culture conversion despite more than 18.8 months of treatment using five effective second-line MDR-TB drugs. During the study period, ESR levels tended to decrease after 6 months of MDR-TB treatment. However, there was no notable trend of changing of inflammatory markers, except that the baseline ESR level was highest in patient 4 who was successfully treated without recurrence.

**Figure 1:**
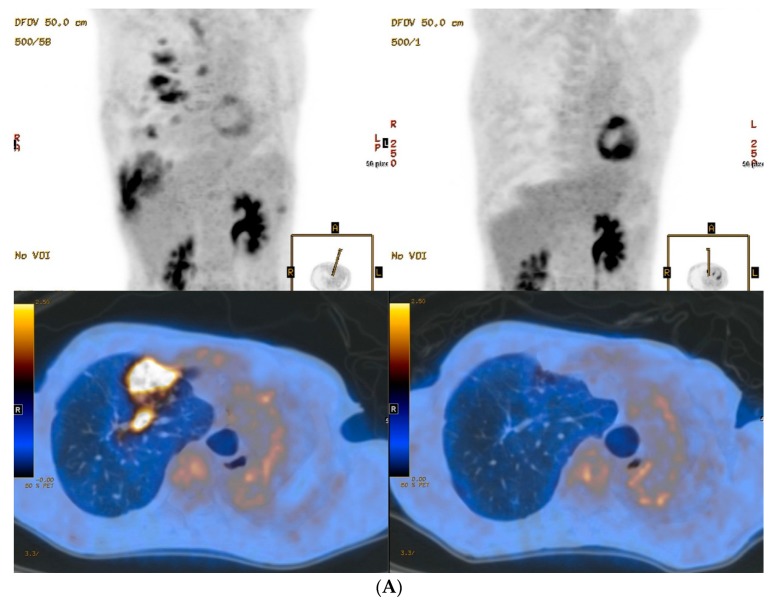

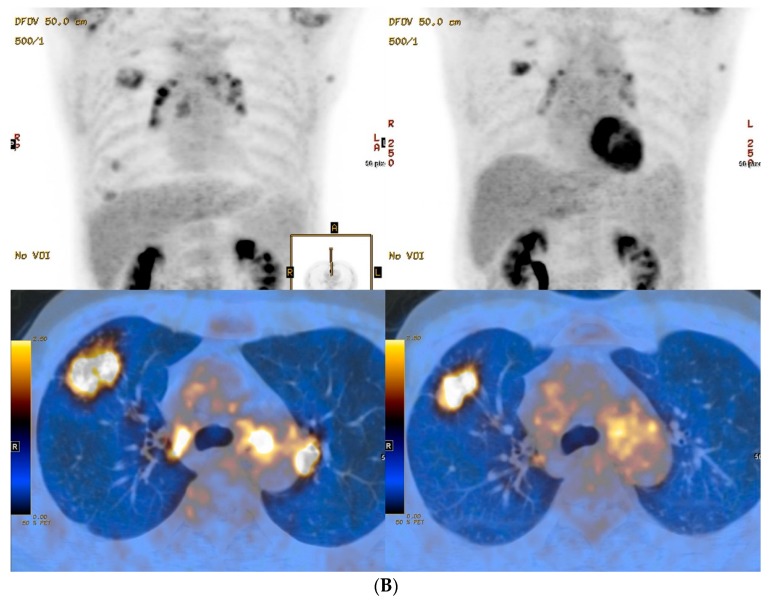
PET/CT images of lungs from two MDR-TB patients. Baseline (left column) and 12 months follow-up (right column) maximum-intensity projection and transverse fusion PET/CT images of patient 2 (**A**) and patient 4 (**B**). (**A**) Images of patient 2 show multiple hypermetabolic lesions in the right lung at baseline that disappeared in follow-up images despite persistent disease. (**B**) Images of patient 4 show multiple hypermetabolic lesions in the right lung at baseline that improved but were persistent in follow-up images irrespective of negative culture conversion and treatment success. Total metabolic lung volume (92.3 cm^3^) and total lung glycolysis (307.3 cm^3^) in patient 2 using a baseline PET/CT were much higher than for patient 4 (4.6 cm^3^ and 14.1 cm^3^, respectively).

The baseline PET/CT scan was examined on the day of starting MDR-TB treatment using a second line anti-TB drug regimen in all study patients. Among baseline PET parameters, both highest SUV_max_ (range, 4.6–7.8) and mean SUV_avg_ (range, 3.0–4.1) appeared to be similar between patients. On the contrary, wide ranges for both TMLV (4.6–92.3 cm^3^) and TLG (14.1–307.3 cm^3^), volume-based PET parameters, were found among the patients. All baseline PET parameters of patients with positive acid-fast bacilli test on the sputum smear (patients 1 and 2) were higher than those of patients with negative acid-fast bacilli tests (patients 3 and 4). Notably, the highest TMLV and TLG parameters were observed in patient 2, who failed culture conversion, compared to the parameters in the three patients with treatment success (Table 2, Figure 1). However, this relationship was not observed at baseline for other SUV-based PET parameters, such as highest SUV_max_ and mean SUV_avg_. All quantitative PET parameters, which were obtained at the start of MDR-TB treatment and 6 and 12 months after treatment, decreased from baseline in all four patients. Notably, there were no significant residual hypermetabolic lesions on follow-up PET at 12 months after treatment in patient 2, who failed culture conversion. Consequentially, high baseline TMLV and TLG levels seemed to be associated with treatment failure, and no other PET parameters at baseline or follow-up were associated with treatment failure in our patients.

## 4. Discussion

In the present study, the most notable finding was that baseline levels of TMLV and TLG in patient 2, the only patient who failed culture conversion, were remarkably higher than those of the other patients with MDR-TB. Moreover, patients 1 and 2, who initially had positive acid-fast bacilli smear results and reflected higher disease burden, had higher baseline levels of TMLV and TLG than patients 3 and 4, who were negative for acid-fast bacilli. These findings suggest that the pretreatment ^18^F-FDG PET parameters of volume-based TMLV and TLG may be helpful for evaluating disease activity and assessing MDR-TB treatment outcome.

To date, several studies have attempted to predict therapeutic outcomes of TB using various PET parameters [11,12,13,14]. For example, Stelzmueller et al. had evaluated values of ^18^F-FDG PET/CT at the initial evaluation and during follow-up in 35 patients with pulmonary or extra-pulmonary TB [13]. In their study, however, they had primarily compared abnormal findings in PET and CT images during the follow-up period. Demura et al. had also shown that post-treatment SUV uptake was remarkably decreased in 14 patients with pulmonary mycobacteria disease, which included eight cases of pulmonary TB and six cases of nontuberculous mycobacterial pulmonary disease [11]. However, they had only identified a trend of decreasing SUV values during monitoring, and no associated parameter with treatment failure was evaluated. Chen et al. have identified that quantitative changes in SUV values after 2 months of treatment were associated with the outcomes of 28 MDR-TB cases, including four patients with treatment failure [16]. However, the trend of decreasing SUV values after 2 months of treatment observed in failed patients was similar in successfully treated patients. Most notably, the aforementioned previous studies had not evaluated volume-based ^18^F-FDG uptake measurements, such as TMLV and TLG, to assess MDR-TB treatment response. Thus, in these contexts, our data highlight the importance of baseline PET parameters, such as TMLV or TLG, that reflect the metabolism of entire lesions, rather than SUV values or follow-up PET parameters, especially for assessing MDR-TB treatment outcomes.

Another notable finding in our data was that all PET parameters such as SUV, TMLV, and TLG consistently decreased in all MDR-TB patients within 12 months of treatment, even in the patient who failed culture conversion. These results suggest that it may be difficult to assess the treatment response based only on the decreasing PET parameters. A potential explanation for this phenomenon is that ^18^F-FDG uptake reflects the metabolic activity of secondary inflammatory cells rather than the actual microbial burden of TB. Although the metabolic activity of secondary inflammatory cells may be correlated with the actual microbial burden of TB before treatment, our findings suggest that secondary lung inflammation could be improved in accordance with decreasing bacterial burden after antibiotic therapy, irrespective of complete eradication of the TB bacilli.

There were several limitations in this study. First, the major limitation of our study is the small sample size, including only four patients, which limits the statistical power of our data for a definite conclusion. In addition, only one of four patients failed treatment. Second, the baseline characteristics of the patients might be the confounding factors for our results. However, we could not find and correct the potential confounding effects due to the small number of subjects. Therefore, our preliminary findings should be tested on the larger numbers of MDR-TB patients for generalizability.

## 5. Conclusions

The present study suggests that pretreatment volume-based ^18^F-FDG PET/CT lung parameters may be useful for evaluating disease activity and assessing therapeutic response in patients with MDR-TB in the lung. Interim ^18^F-FDG PET/CT does not seem to be necessary for assessing the final therapeutic response. Our preliminary results warrant further larger-sized studies.

## 6. Ethics Approval and Consent to Participate

All procedures performed in studies involving human participants were in accordance with the ethical standards of the institutional and/or national research committee and with the 1964 Helsinki Declaration and its later amendments or comparable ethical standards. The Institutional Review Board of Samsung Medical Center is the local ethics committee that reviewed and approved the protocol. Informed consent was obtained from all individual participants included in the study.

## Figures and Tables

**Table 1:** Baseline characteristics of patients at diagnosis of multidrug-resistant tuberculosis (MDR-TB).

Characteristics	Patient No.			
	1	2	3	4
Age, years	35	59	42	61
Sex	Female	Female	Female	Male
AFB smear	2+	2+	-	-
Body mass index, kg/m^2^	20.6	15.1	24.1	25.0
Previous history of TB	TB LN	MDR-TB	-	DS-TB
Time from treatment completion of previous TB to inclusion of current study	13 years	8 years	-	30 years
Duration of previous TB treatment	9 months	23 months	-	NA
Regimens of previous TB treatment	1st line anti-TB drugs	EMB PZA KM MFX PTO CS PAS	-	1st line anti-TB drugs
Previous lung surgery	-	pneumonectomy	-	-
Time from starting MDR-TB treatment to PET/CT scan	0 day	0 day	0 day	0 day
Cavity size	12 mm	17 mm	31 mm	-
WBC count, 10^3^/uL	4510	7350	5720	4840
ESR, mm/hr	21	66	14	88
CRP, mg/dL	0.11	0.08	0.06	0.28
Albumin, g/dL	4.3	4.6	4.3	4.0

AFB, acid-fast bacilli; MDR, multidrug resistant; TB, tuberculosis; LN, lymphadenitis; DS, drug susceptible; EMB, ethambutol; PZA, pyrazinamide; KM, kanamycin; MFX, moxifloxacin; PTO, prothionamide; CS, cycloserine; PAS, para-aminosalicylic acid; PET/CT, positron emission tomography/computed tomography; WBC, white blood cell; ESR, erythrocyte sedimentation rate; CRP, C-reactive protein; NA, not available.

**Table 2:** Treatment outcomes for MDR-TB and serial changes of positron emission tomography/computed tomography (PET/CT) parameters.

Patient No.	Drug Resistance	Treatment Regimens	Outcome	PET/CT	Highest SUV_max_	Mean SUV_avg_	TMLV (cm^3^)	TLG (cm^3^)
1	INH, RFP, EMB, PAS	PZA, KM, MFX, PTO, CS	Success	Baseline	7.8	4.1	31.4	129.8
				6 mo	4.8	3.1	2.0	6.1
				12 mo	2.3	NA	NA	NA
2	INH, RFP, FQ, EMB, PAS	KM, PTO, CS, LZD, BDQ, CFZ	Failure	Baseline	6.6	3.3	92.3	307.3
				6 mo	3.4	2.9	0.4	1.3
				12 mo	NV	NA	NA	NA
3	INH, RFP, FQ, EMB	EMB, KM, PTO, CS, LZD, BDQ	Success	Baseline	5.4	3.1	11.0	34.3
				6 mo	4.6	3.0	2.0	5.9
				12 mo	2.3	NA	NA	NA
4	INH, RFP, EMB, PAS	PZA, KM, MFX, PTO, CS	Success	Baseline	4.6	3.0	4.6	14.1
				6 mo	5.6	3.6	6.3	22.7
				12 mo	4.7	3.3	3.7	12.2

Abbreviations: MDR-TB, multidrug-resistant tuberculosis; AFB, acid-fast bacilli; INH, isoniazid; EMB, ethambutol; RFP, rifampin; FQ, fluoroquinolone; PZA, pyrazinamide; KM, kanamycin; MFX, moxifloxacin; PTO, prothionamide; CS, cycloserine; PAS, para-aminosalicylic acid; LZD, linezolid; BDQ, bedaquiline; CFZ, clofazimine; PET/CT, positron emission tomography/computed tomography; SUV, standardized uptake value; TMLV, total metabolic lung volume; TLG, total lung glycolysis; NA, not applicable; NV, non-visualized; mo: month.
